# Exploring cognitive and neuroimaging profiles of dementia subtypes of individuals with dementia in the Democratic Republic of Congo

**DOI:** 10.3389/fnagi.2025.1552348

**Published:** 2025-02-12

**Authors:** Jean Ikanga, Saranya Sundaram Patel, Megan Schwinne, Caterina Obenauf, Emmanuel Epenge, Guy Gikelekele, Nathan Tshengele, Immaculee Kavugho, Samuel Mampunza, Lelo Mananga, Charlotte E. Teunissen, Julio C. Rojas, Brandon Chan, Argentina Lario Lago, Joel H. Kramer, Adam L. Boxer, Andreas Jeromin, Emile Omba, Alvaro Alonso, Alden L. Gross

**Affiliations:** ^1^Department of Rehabilitation Medicine, Emory University School of Medicine, Atlanta, GA, United States; ^2^Department of Psychiatry, School of Medicine, University of Kinshasa and Catholic University of Congo, Kinshasa, Democratic Republic of Congo; ^3^One Rehab, Dallas, TX, United States; ^4^Department of Biomedical Informatics, School of Medicine, Emory University, Atlanta, GA, United States; ^5^Department of Psychology, University of Tennessee, Knoxville, Knoxville, TN, United States; ^6^Memory Clinic of Kinshasa, Kinshasa, Democratic Republic of Congo; ^7^Department of Neurology, University of Kinshasa, Kinshasa, Democratic Republic of Congo; ^8^Neurochemistry Laboratory, Department of Clinical Chemistry, Amsterdam Neuroscience, Neurodegeneration, Amsterdam University Medical Centers, Vrije Universitiet, Amsterdam, Netherlands; ^9^Department of Neurology, Memory and Aging Center, Weill Institute for Neurosciences, University of California, San Francisco, San Francisco, CA, United States; ^10^ALZpath, Inc., San Francisco, CA, United States; ^11^Department of Epidemiology, Rollins School of Public Health, Emory University, Atlanta, GA, United States; ^12^Department of Epidemiology, Johns Hopkins Bloomberg School of Public Health, Baltimore, MD, United States

**Keywords:** dementia, Democratic Republic of the Congo, cognition, neuroimaging, biomarkers

## Abstract

**Objective:**

The 2024 Alzheimer’s Association (AA) research diagnostic criteria for Alzheimer’s Disease (AD) considers fluid biomarkers, including promising blood-based biomarkers for detecting AD. This study aims to identify dementia subtypes and their cognitive and neuroimaging profiles in older adults with dementia in the Democratic Republic of Congo (DRC) using biomarkers and clinical data.

**Methods:**

Forty-five individuals with dementia over 65 years old were evaluated using the Community Screening Instrument for Dementia and the informant-based Alzheimer’s Questionnaire. Core AD biomarkers (Aβ42/40 and p-tau181) and non-specific neurodegeneration biomarkers (NfL, GFAP) were measured in blood plasma. Neuroimaging structures were assessed using magnetic resonance imaging (MRI). Dementia subtypes were determined based on plasma biomarker pathology and vascular markers. Biomarker cutoff scores were identified to optimize sensitivity and specificity. Individuals were stratified into one of four dementia subtypes—AD only, non-AD vascular, non-AD other, or mixed – based on combinations of abnormalities in these markers.

**Results:**

Among the 45 individuals with dementia, mixed dementia had the highest prevalence (42.4%), followed by AD-only (24.4%), non-AD other dementia (22.2%), and non-AD vascular dementia subtypes (11.1%). Both cognitive and neuroimaging profiles aligned poorly with biomarker classifications in the full sample. Cognitive tests varied across dementia subtypes. The cognitive profile of the AD-only and mixed groups suggested relatively low cognitive performance, while the non-AD and other groups had the best scores on average.

**Conclusion:**

Consistent with studies in other settings, our preliminary findings suggest that neurodegenerative plasma biomarkers may help to identify dementia subtypes and provide insight into cognitive and neuroimaging profiles among older adults in the DRC.

## Introduction

Alzheimer’s disease (AD) is the most common neurodegenerative disease, with pathology characterized by the accumulation of amyloid-beta (Aβ) plaques and neurofibrillary tangles composed of hyperphosphorylated tau protein (Balasaheb Chavan et al., 2023). With the advancements in assay technology, plasma biomarkers have increasingly been shown to have potential for the detection and monitorization of AD, increasing accessibility beyond catchment areas of major medical centers (Dimtsu Assfaw et al., 2024; Palmqvist et al., 2024). Current revised 2024 Alzheimer’s Association (AA) criteria distinguish three broad categories of AD fluid biomarkers related to AD pathogenesis: (1) core AD fluid biomarkers [the CSF ratio of amyloid-β (Aβ42/40), phosphorylated and secreted AD tau (p-tau 217, p-tau-181, and p-tau 231)], (2) non-specific biomarkers involved in other neurodegenerative pathology, including neurofilament light (NfL) and glial fibrillary acidic protein (GFAP), and (3) biomarkers of non-AD pathology (vascular brain injury, alpha-synuclein [αSyn]) (Jack et al., 2024). Identifying plasma biomarkers for underlying pathologies of dementia can especially benefit prodromal or pre-clinical stages, for which current and emerging disease-modifying therapies are more likely to be effective (Ashton et al., 2020).

Using blood biomarkers known to provide early indication of a disease may facilitate more timely diagnosis for patients exhibiting early symptoms, particularly in early-onset and atypical presentations. Blood-based biomarkers in AD are associated with both early indicators of cognitive decline and longitudinal cognitive outcomes (Dimtsu Assfaw et al., 2024). For example, lower plasma Aβ42/Aβ40 ratios correlate with higher amyloid plaque burden and cognitive impairment and can be detected in preclinical disease stages (Nakamura et al., 2018), making it useful for early diagnosis and tracking disease progression (Palmqvist et al., 2019). Elevated levels of p-tau181 and p-tau217 are observed in AD, serving as indicators of both early and late stages of AD (Janelidze et al., 2020; Karikari et al., 2020). Plasma p-tau increases in early symptomatic stages, aligning with clinical transition from mild cognitive impairment (MCI) to AD dementia (Thijssen et al., 2020). NfL is a marker of axonal damage; while less specific, elevated levels of NfL reflect more widespread neuronal damage (Mattsson et al., 2017). GFAP reflects astrocytic activation and neuroinflammation, with increased levels observed in AD. GFAP may be used to complement other biomarkers to enhance diagnostic accuracy, particularly in advanced stages (Oeckl et al., 2022). However, additional data are still needed to demonstrate the utility and validity of blood biomarkers in diverse clinical cohorts and to accurately detect disease profiles, particularly given overlapping symptom profiles across different pathologies. For example, vascular damage and protein alterations are present in most forms of dementia, which adds a layer of uncertainty to diagnosis given the potential for mixed dementia pathology (Dodge et al., 2017). In vascular disease, NfL may also be elevated as axonal injury can be seen in cerebrovascular disease. NfL concentrations reflect acute and chronic cerebrovascular injury, which is useful for both early detection and monitoring progression (Mattsson et al., 2019). In addition to plasma biomarkers, structural neuroimaging may also provide important additional diagnostic data. Specifically, the entorhinal cortex and hippocampal regions are particularly affected in the early stages of AD (Balasaheb Chavan et al., 2023; Igarashi, 2023). Hippocampal volume loss is a feature differentiating AD dementia from other dementias, such frontotemporal dementia (FTD) and vascular dementia (VaD), and is closely linked to the course of AD (Killiany et al., 2002; MacLin et al., 2019).

A significant caveat is that most research involving neurodegenerative biomarkers in AD primarily have been conducted using Western cohorts. Studies have shown that CSF biomarkers, such as reduced levels of Aβ42 and p-tau, correlate with AD pathology and can aid in distinguishing AD from other forms of dementia (Van Harten et al., 2021), but less is known about the biomarker and neuroimaging parameters and profiles in diverse populations, particularly in Sub-Saharan African (SSA) populations. This study expands on previous biomarker research by focusing exclusively on SSA populations, where the prevalence and profiles of dementia subtypes remain underexplored. Unlike previous studies, the current study focuses on the variability in biomarker expression and neuroimaging findings in SSA, contributing to the small literature on this topic in this underrepresented region.

The current study aims to explore dementia subtypes based on blood-based biomarkers and vascular factors, and their neuroimaging and cognitive profiles in adult individuals with clinical dementia in Kinshasa, Democratic Republic of Congo (DRC), in SSA. We expected that there is higher prevalence of participants with non-AD pathologies compared to those with AD dementia subtype. Based on previous studies that have linked amyloid-β deposition, tau protein, and neurodegeneration (NfL) accumulations with impairments in language, learning and memory, and executive function, we hypothesized that the cognitive patterns aligning with neurodegenerative biomarkers are characterized by deficits in these cognitive domains (Mattsson et al., 2017; Nakamura et al., 2018; Palmqvist et al., 2019; Slot et al., 2019; Janelidze et al., 2020; Karikari et al., 2020; Thijssen et al., 2020; Oeckl et al., 2022). The current study aims to focus on well-defined dementia cases, ensuring robust differentiation between probable AD dementia and healthy controls (HC); thus, cases with MCI were not included. Focusing on probable AD dementia provides a clearer understanding of advanced disease stages and reduces diagnostic ambiguities associated with MCI, where conversion to dementia is not guaranteed. Similarly, since amyloid-β deposition and tau protein accumulation in the brain are associated with atrophy in the hippocampus, temporal lobe, medial temporal, and entorhinal cortex, we expected that the neuroimaging patterns that align with neurodegenerative biomarkers are characterized by atrophy in these structures (Killiany et al., 2002; MacLin et al., 2019; Balasaheb Chavan et al., 2023; Igarashi, 2023). A general comparison of cognitive deficits and brain atrophy reveals more severe and distinct patterns of deficits and atrophy in Alzheimer’s disease (AD) participants, followed by those with mixed dementia, and vascular dementia.

## Materials and methods

### Study population

Participants of this study are community-dwellers from Kinshasa/DRC diagnosed with dementia and selected from a prevalence study of dementia (Ikanga et al., 2023b). Study design details have been published previously (Ikanga et al., 2023b). Briefly, participants were included if they were at least 65 years or older, had a family member or close friend to serve as an informant, and fluent in French or Lingala. We excluded individuals who had history of schizophrenia, neurological, or other medical conditions potentially affecting the central nervous system (CNS), yielding a sample of 1,432 eligible participants. To establish neurological status in the absence of established diagnostic criteria for AD in Sub-Saharan Africa (SSA), we screened eligible participants using the Alzheimer’s Questionnaire (AQ) (Malek-Ahmadi and Sabbagh, 2015) and the Community Screening Instrument for Dementia (CSID) (Hall et al., 2000; Imarhiagbe et al., 2005). The AQ assesses activities of daily living and symptoms of AD in participants (Hall et al., 2000). The CSID Questionnaire, used in several SSA dementia studies (Akinyemi et al., 2014; Farombi et al., 2016; Ogbimi et al., 2023), was used to screen cognitive abilities.

**FIGURE 1:**
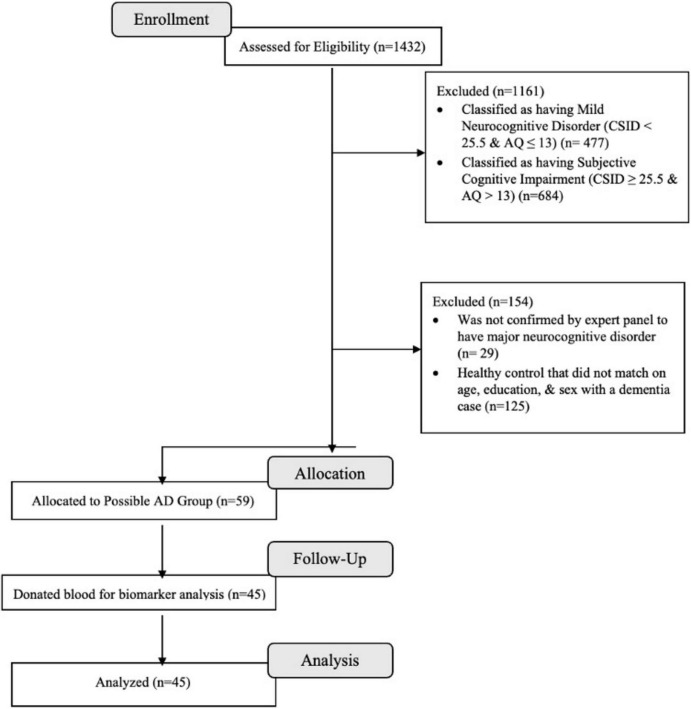
Flow chart of recruitment status from those assessed for eligibility at enrollment (*n* = 1,432) to the individuals that were allocated to the dementia and donated blood for biomarkers (*n* = 45).

Based on cognitive and functional deficits per the Diagnostic and Statistical Manual of Mental Disorders, Fifth Edition, Text Revision (DSM-5-TR) diagnostic criteria (American Psychiatric Association, 2013), we classified eligible participants using CSID cut-offs from a previous study conducted in Congo-Brazzaville, the closest city from Kinshasa (Guerchet et al., 2010). Similar to our prior study (Ikanga et al., 2023b), eligible participants were classified using CSID and AQ scores (see Figure 1) which resulted in 1,161 individuals being excluded based on their having only mild neurocognitive disorder (MND) or subjective cognitive impairment.

A panel consisting of a neurologist (EE), psychiatrist (GG), and neuropsychologist (JI) reviewed screening tests, clinical interview, and neurological examination of 271 subjects, of whom 59 from 88 were confirmed with a diagnosis of dementia and 58 from 183 were considered HC. Of these 117 participants, 29 refused to provide blood samples, leaving 85 participants (75%) in whom plasma biomarkers were obtained (45 dementia and 40 HC) who were matched on age, education, and sex. For the present analysis, only participants with dementia were included (see Figure 1). Written informed consent was obtained prior to participants’ undergoing any study procedures. Participants were financially compensated for their time. The procedures were approved by the Ethics Committee/Institutional Review Boards of the University of Kinshasa and Emory University.

### Procedure

Participants underwent a comprehensive clinical evaluation, including cognitive testing, self-report questionnaires, and standard psychiatric and neurological evaluations. Subjects were interviewed to obtain demographic, socioeconomic, and medical history and were subsequently administered cognitive testing with African Neuropsychological Battery (ANB) subtests.

### Measures

#### Plasma biomarkers

Blood samples were drawn at the Medical Center of Kinshasa (CMK) blood laboratory by antecubital venipuncture into dipotassium ethylene diamine tetra acetic acid (K_2_ EDTA) tubes. Samples were centrifuged within 15 min at 1,800 g house temperature, and 5 mL of plasma was aliquoted into 0.5 mL polypropylene tubes and stored initially at −20°C for less than a week and stored in a −80°C freezer for longer term storage at a CMK laboratory. These aliquots were shipped frozen on dry ice to Emory University for storage and then to University of California San Francisco (UCSF) for measurements.

Plasma biomarker concentrations were measured using commercially available Neurology 4-PLEX E (Aβ40, Aβ42, NfL, and GFAP; lot #503819), P-Tau181 (P-Tau181 v2; lot #503732), IL-1b (lot #503806) and IL-10 (IL-10 2.0, lot #503533) Quanterix kits on the Simoa HD-X platform (Billerica, MA) at UCSF. P-tau217 was measured using the proprietary ALZpath pTau-217 CARe Advantage kit (lot #MAB231122, ALZpath, Inc.) on the Simoa HD-X platform. The instrument operator was blinded to clinical variables. All analytes were measured in duplicate, except for IL-1b, which was measured as a singlicate due to low sample availability (Kirmess et al., 2021; West et al., 2021; Hu et al., 2022). For Aβ40, Aβ42, NfL, and GFAP, all samples were measured above the lower limit of quantification (LLOQ) of 1.02 pg/mL, 0.378 pg/mL, 0.4 pg/mL and 2.89 pg/mL, respectively. The average coefficient of variation (CV) for Aβ40, Aβ42, NfL, and GFAP were 6.0, 6.5, 5 and 4.6%, respectively. For IL-1b and IL-10, the LLOQ were 0.083 and 0.021 pg/mL, respectively. The average CV for IL-10 was 6.1%. For P-tau217 the LLOQ was 0.024 pg/mL and the average CV was 19.8%.

#### Neuroimaging

All subjects were imaged on a 1.5 Tesla MRI unit (Siemens, Magneton Sonata) scanner at HJ Hospitals in Kinshasa using the same standardized imaging acquisition protocol based on the Alzheimer’s Disease Research Center (ADRC) protocol of Emory University (Ikanga et al., 2023a). This consisted of sagittal volumetric T1-weighted (MPRAGE), coronal T2-weighted, and axial diffusion-weighted, T2-weighted, and T2-FLAIR sequences. Typical acquisition parameters for the MPRAGE sequence were TR = 2,200 ms, minimum full TE, TI = 1,000 ms, flip angle = 8°, FOV = 25 cm, with a 192 × 184 acquisition matrix, yielding a voxel size of approximately 1.25 × 1.25 × 1.2 mm. The standard MPRAGE, with a shorter TE of 4 ms and a TI of 1,000 ms, was used to achieve better contrast and image quality.

Images were reviewed by a subspecialty certified neuroradiologist (AMS) with 14 years of experience. White matter hyperintensities were graded according to the Age-Related White Matter Changes (ARWMC) scale (Wahlund et al., 2001), The WMH were graded on the T2 FLAIR images, as they are most readily visible and graded on that sequence. The number of chronic brain parenchymal microhemorrhages were recorded. MPRAGE images were reoriented into the oblique coronal plane orthogonal to the principal axis of the hippocampal formation, and medial temporal lobe atrophy (MTLA) (Claus et al., 2017) and entorhinal cortex atrophy (EriCa) (Enkirch et al., 2018) scores were assessed. Finally, the presence or absence of any additional abnormalities was noted, and patients were excluded if neuroimaging evidence indicated an etiology other than probable AD (e.g., presence of a brain tumor).

#### Quantitative volumetric analysis using Freesurfer

The 3D T1 images were segmented using Freesurfer (v.6, MGH, MA), which includes a full processing stream for MR imaging data that involves skull-stripping, bias field correction, registration, and anatomical segmentation as well as cortical surface reconstruction, registration, and parcelation. Regional brain volume for both cortical and subcortical brain regions were calculated. The left and right hippocampal volume were averaged. Interindividual variation in head size were accounted for in further statistical analysis by controlling for the effects of the total intracranial volume.

### Determination of dementia subtypes

Dementia subtypes were determined based on the plasma biomarkers (Aβ_42/40,_ p-tau_181_, NfL, GFAP), alongside vascular markers, hemoglobin A1c (HbA1c), blood pressure, and total cholesterol. Given lack of established AD biomarker thresholds in the DRC/SSA, determination of biomarker thresholds was informed by prior analysis conducted by Ikanga et al. (2024). These analyses utilized logistic regression models to assess the relationship between individual biomarkers and neurological status (healthy or suspected AD) (Ikanga et al., 2023a). Receiver operating characteristic (ROC) curve analyses were conducted to evaluate the diagnostic accuracy of the biomarkers, calculating the area under the curve (AUC) for each. Biomarker cutoff scores were defined by maximizing sensitivity and specificity, determined by the highest Youden’s Index, to optimize the classification of neurological status in this population. Thresholds for vascular markers, HbA1c, hypertension, and hypercholesterolemia, were sourced from existing literature (Kowall and Rathmann, 2013; Whelton et al., 2017; Nantsupawat et al., 2019; Li et al., 2021). Individuals with elevated HbA1c (≥ 6.5%), blood pressure (systolic ≥ 130 mmHg or diastolic ≥ 80 mmHg), or total cholesterol (≥ 200 mg/dL) were deemed to have dementia of potential vascular etiology. Subsequently, individuals were classified into one of four dementia subtypes—AD only, non-AD vascular, non-AD other, or mixed—based on their presence or absence of these biomarkers (Table 1).

**TABLE 1:** Organization of biomarkers into pathological subtypes of dementia utilizing (Ikanga et al., 2024 ; Hu et al., 2022) threshold for core AD biomarkers.

Biomarker	Threshold	Pathological type			
		**AD only**	**Non-AD vascular**	**Non-AD other**	**Mixed**
**Core AD biomarkers**					
Decreased Aβ_42/40_	≤0.061 pg/mL	Present	Absent	Absent	Present
Increased p-tau_181_	≥4.50 pg/mL				
**Non-specific AD biomarker**					
Increased GFAP	≥176.0 pg/mL	Optional	Present	Optional	Optional
Vascular markers*					
High HbA1c	≥6.5%	Absent	Present	Optional	Present
Hypertension	SBP ≥ 130 or DBP ≥ 80 mmHg				
Hypercholesterolemia	TC ≥ 200 mg/dL				

*Note-in merged rows, if a biomarker is required to be present, only one, but not limited to one biomarker needs to be present. However, all biomarkers required to be absent must be absent; for example, AD only subtype requires either Decreased Aβ_42/40_, Increased p-tau_181_, or both. Increased GFAP is optional, but all vascular markers must be absent. AD, Alzheimer’s Disease; Aβ_42/40_, ratio of amyloid beta 42 and amyloid beta 40; p-tau_181_, phosphorylated tau protein 181; NfL, neurofilament light chain; GFAP, glial fibrillary acidic protein; HbA1c, hemoglobin A1c; SBP/DBP, systolic/diastolic blood pressure; TC, total cholesterol.

### Statistical analyses

Descriptive statistics were employed to summarize the data, with continuous variables reported as means and standard deviations, and categorical variables reported as frequencies and row percentages. We used linear regression models to compare differences in demographics, biomarkers, vascular markers, neuroimaging measures, and cognitive tests by dementia subtype. Models were adjusted for age, gender, years of education, total intracranial volume (for neuroimaging variables), and Geriatric Depression Scale (GDS) score. Subsequently, Dunn’s *post-hoc* test for pairwise comparisons was conducted to explore differences in neuroimaging and cognitive assessment measure between biomarker-defined dementia subtypes. Results were evaluated with a significance set at *p* < 0.05. Statistical analysis was conducted using R version 4 statistical software.

## Results

Demographic data, neurodegenerative plasma biomarkers, vascular markers, neuroimaging, and cognitive characteristics are presented in Table 2. The sample comprised 45 clinically adjudicated dementia participants, of whom 20 (44%) were males, with an average age of 73.8 years (SD = 8 years) and an average of 7.4 years of education (SD = 5 years). Clinically, the sample exhibited high symptoms of depression (GDS = 7.5), and 58% of the participants had clinical hypertension (see Table 2).

**TABLE 2:** Characteristics of the study sample.

Characteristic, μ (σ)	Clinical dementia (*n* = 45)
Age (years)	73.8 (8)
Male (n,%)	20 (44%)
Education (years)	7.4 (5)
GDS score	7.5 (3.5)
**Biomarkers**	
Aβ_42/40_	0.06 (0.03)
p-tau 181 (pg/mL)	3.0 (2)
NfL (pg/mL)	62.7 (41)
GFAP (pg/mL)	241.0 (144)
**Vascular markers**	
HbA1c (%)	5.6 (0.7)
Hypertension (n,%)	26 (58%)
High cholesterol (n,%)	1 (2%)
**Neuroimaging measures**	
Intracranial volume (mm^3^)	1433637 (277941)
Left hippocampal volume (mm^3^)	2970 (535)
Right hippocampal volume (mm^3^)	2973 (573)
Left entorhinal cortex volume (mm^3^)	1525 (573)
Right entorhinal cortex volume (mm^3^)	1642 (568)
White matter hyperintensity	70.0 (2.6)
Microhemorrhage	0.69 (1.5)
Mesial temporal atrophy score	2.3 (1.1)
Entorhinal cortex atrophy score	1.7 (0.78)
**Cognitive tests**	
CSID	19.6 (5.6)
AQ	19.3 (4.0)
African naming test	15.5 (7.2)
ALMT trial 1	2.6 (1.7)
AVMT trial 1	1.3 (1.6)
ALMT trial 3	4.0 (1.9)
AVMT trial 3	1.9 (1.9)
ALMT recall	0.31 (0.6)
AVMT recall	1.0 (1.7)
Proverb test	2.5 (2.2)
Card game wins	21.7 (7.0)

*GDS, Geriatric Depression Score; NfL, Neurofilament Light; GFAP, Glial Fibrillary Acidic Protein; CSID, Community Screening Instrument for Dementia; AQ, Alzheimer’s Questionnaire; ALMT, African List Memory Test; AVMT, African Visuospatial Memory Test.

Table 3 presents the dementia subtype defined by neurodegenerative plasma biomarkers using Ikanga and colleagues’ threshold (Ikanga et al., 2024). As anticipated, there is a higher prevalence of mixed dementia, followed by AD-only, non-AD other dementia, and non-AD vascular dementia patterns.

**TABLE 3:** Clinical dementia subtype based on biomarker patterns.

Threshold	Biomarker patterns of participants with dementia, n (%) (*n* = 45)			
	**AD only pattern**	**Non-AD other pattern**	**Non-AD vascular pattern**	**Mixed pattern**
Ikanga et al. (2024)	11 (24.4%)	10 (22.2%)	5 (11.1%)	19 (42.4%)

Table 4 presents the cognitive profiles for each dementia subtype based on the cutoff criteria established by Ikanga et al. (2024). We excluded the vascular dementia subtype from these analyses due to the small sample size (only five participants). Cognitively, there were no clinically or statistically significant differences between the dementia subtypes. The cognitive profiles do not align well with biomarker-based dementia subtypes.

**TABLE 4:** Cognitive profile for dementia subtypes.

Cognitive test	Dementia subtype, μ (σ)			
	**AD only** **(*n* = 11)**	**Non-AD other** **(*n* = 10)**	**Mixed** **(*n* = 19)**	***p*-value**
African Naming Test	12.6 (6.44)	19.5 (5.85)	14.9 (7.67)	0.099
ALMT Trial 1	2.22 (0.97)	3.30 (1.34)	2.89 (1.91)	0.32
AVMT Trial 1	1.11 (1.27)	1.90 (1.66)	1.39 (1.82)	0.50
ALMT Trial 3	3.22 (1.39)	4.90 (1.66)	4.22 (2.02)	0.16
AVMT Trial 3	1.11 (1.05)	2.60 (2.50)	2.11 (1.84)	0.11
ALMT Recall	0.00 (0.00)	0.30 (0.67)	0.50 (0.79)	0.22
AVMT Recall	0.22 (0.44)	1.70 (2.41)	1.11 (1.60)	0.15
Proverb Test	2.00 (1.22)	4.00 (3.37)	2.11 (1.64)	0.056
Card Game Wins	23.8 (8.17)	21.9 (6.88)	16.4 (4.93)	0.76

Table 5 presents the neuroimaging profile for each dementia subtype based on Ikanga and colleagues’ threshold (Ikanga et al., 2024). As in the previous analyses, we did not include the vascular dementia subtype because there are only five participants in this subtype. The neuroimaging profiles do not align well with biomarker-based dementia subtypes. The AD group showed reduced scores in many neuroanatomical structures compared to other dementia subtypes. There was a statistical difference in left hippocampal volume between various dementia subtypes, mostly between AD-only and non-AD other subtypes, and between AD-only and mixed subtypes. There was a trend in terms of microhemorrhage between dementia subtypes (see Table 5).

**TABLE 5:** Neuroimaging profile for dementia subtypes.

Neurological measure	Dementia subtype, mean (SD)			
	**AD only** **(*n* = 11)**	**Non-AD other** **(*n* = 10)**	**Mixed** **(*n* = 19)**	***p*-value**
Intracranial volume (mm^3^)	1,595,574 (506,956)	1,419,302 (1,62,980)	1364517 (139986)	0.11
Left hippocampus (mm^3^)*^†^	2,523 (355)	3,122 (408)	3142 (611)	0.006
Right hippocampus (mm^3^)	2,596 (210)	3,011 (564)	3122 (730)	0.13
Left entorhinal cortex (mm^3^)	1,248 (431)	1,696 (504)	1596 (620)	0.12
Right entorhinal cortex (mm^3^)	1,433 (504)	1,732 (443)	1750 (707)	0.24
White matter hyperintensity	70.3 (2.97)	71.0 (2.39)	69.3 (2.62)	0.36
Microhemorrhage	0 (0)	5 (50%)	3 (16%)	0.053
Mesial temporal atrophy score	2.78 (0.83)	2.10 (0.99)	1.77 (1.01)	0.081
Entorhinal cortex atrophy score	1.78 (0.83)	1.60 (0.70)	1.54 (0.88)	0.66

*Statistically significant difference between AD-only and non-AD other subtypes.

^†^Statistically significant difference between AD-only and mixed subtypes. Presence of microhemorrhages is a dichotomous variable, represented as n (%).

## Discussion

This study primarily aimed to evaluate the feasibility of using neurodegenerative plasma biomarkers to characterize dementia subtypes and describe their cognitive and neuroimaging profiles in a novel sample of older adults with clinical dementia in SSA. Experts have reported gaps in neuropsychological testing instruments, diagnostic procedures, fluid biomarkers, and neuropathological correlative studies. This exploratory study aimed to address the gap in plasma biomarkers in the DRC/SSA.

Despite the absence of a gold standard threshold for neurodegenerative fluid biomarkers in the DRC/SSA, we investigated the cognitive and neuroimaging profiles of adults with dementia in the DRC/SSA, using plasma neurodegenerative biomarkers. We found a high prevalence of mixed, followed by AD only, non-AD other dementia, and non-AD vascular dementia patterns, despite cultural, racial, and geographic differences. These results contrast with Western findings, which indicate that the most prevalent dementias among older adults (65 years and over) are Alzheimer’s Disease (60–80% of cases), vascular dementia (10–20% of cases), mixed dementia (5–15% of cases), and other dementias, such as dementia with Lewy bodies (2–5% of cases), dementia associated with Parkinson’s disease (3.6% of cases), and frontotemporal dementia (2–5% of cases) (Jack et al., 2019; Nichols et al., 2022; No author list, 2023).

These differences in classifying dementia based on clinical and biological markers can be explained by the heterogeneous and continuous nature of Alzheimer’s disease, which is complex to characterize (Young et al., 2018). Clinical adjudication relies on medical history, neuropsychological assessments, cognitive symptoms, and behavioral changes, which can be subjective and prone to variability among clinicians (Liss et al., 2021). Fluid biomarkers can assess specific proteins or molecules, detect biological changes, and provide objective, quantitative measures to refine clinical diagnosis. Therefore, fluid biomarkers can identify pre-symptomatic or prodromal stages of dementia (Giampietri et al., 2022). Thus, there could be changes in the classification of AD prevalence.

Contrary to our second hypothesis, which predicted that the neuroimaging profile would align better with biomarker-based dementia subtypes than with the cognitive profile, we found that neither cognitive nor neuroimaging profile tracked well with plasma biomarkers among clinical dementia participants. The cognitive profile in the AD-only and Mixed groups suggests relatively low cognitive performance, while the Non-AD Other group demonstrated some of the highest average scores. Biological underpinnings may explain some further variance in cognitive profiles, particularly in the AD only group.

Similarly, the neuroimaging profile appeared to track poorly with biomarker classifications among those with clinical dementia. While the Mixed group showed a relatively preserved neuroimaging profile, including the hippocampus, entorhinal cortex, WMH, microhemorrhage, mesial temporal atrophy score, and entorhinal cortex atrophy score, the AD-only biomarker group has significantly lower neurological volumes. Several factors may explain the lack of alignment between neuroimaging, cognitive profiles, and biomarkers. The small sample size may have reduced the ability to detect meaningful differences between subtypes. Additionally, variability in disease stage among participants could obscure relationships, as neuroimaging measures may reflect different phases of pathology. The resolution of neuroimaging techniques may also be insufficient to capture subtype-specific brain changes. Moreover, lifestyle factors that were not controlled for in analysis, such as levels of physical activity or the presence of comorbidities, likely contributed to variability in cognitive and neuroimaging profiles within subtypes. These factors collectively underscore the complexity of linking biomarkers with dementia subtypes.

As noted, this is the first study to explore biomarker-based dementia subtypes and to examine cognitive and neuroimaging profiles in the DRC/SSA using culturally appropriate neuropsychological tests, neuroimaging tools, and fluid biomarkers. The exploratory findings of this study provide evidence of the usefulness of ANB tests and their importance in the algorithm for clinical adjudication of different subtypes of dementia. Our analyses also showed the importance of MRI and plasma biomarkers as diagnostic tools for dementia in SSA/DRC. Overall, the strengths of the current study include the use of culturally validated neuropsychological tests, the ability to collect neuroimaging data in participants who are not familiar with MRI, and plasma biomarkers in a population where there is resistance to donating blood for research due to fear of witchcraft. Lastly, this study used a case-control design to obtain cross-sectional results.

Some limitations of this exploratory study include the modest sample size in this first DRC effort (which may limit the generalizability of the findings and statistical power), given the cost of collection, shipping, and the analyses of plasma biomarkers, MRI scans, and the novel nature of their introduction in the DRC, which created some hesitancy for many potential participants to enroll in the study. Future studies should recruit larger, more diverse cohorts for robust stratified analyses and consider longitudinal designs to explore the temporal relationship between cognitive decline and biomarker changes. These approaches will enhance diagnostic precision and deepen understanding of dementia subtypes in underrepresented populations. Furthermore, limited alignment between cognitive, neuroimaging, and biomarker-based classifications suggests a need for further exploration of methodology. Future studies should also prioritize the recruitment of larger and more diverse cohorts to enable nuanced analyses of sex-based factors in AD and other dementia subtypes within Congolese populations. Growing evidence highlights the differential impact of sex on the risk, progression, and presentation of AD, driven by a combination of biological, hormonal, and sociocultural factors. In the context of the DRC, where sociocultural roles and access to healthcare often vary by gender, such analyses are particularly critical. Larger sample sizes would allow for statistically robust investigations into how sex interacts with biomarkers, cognitive outcomes, and neuroimaging profiles in this population. Additionally, incorporating diverse cohorts reflecting a range of socioeconomic backgrounds, education levels, and urban versus rural living conditions will be essential for identifying unique risk and protective factors. These efforts are vital for developing tailored diagnostic and therapeutic strategies that address the complex interplay between sex and dementia in underrepresented and underserved populations. The discrepancy between cognitive and biomarker-based classifications may reflect limitations in cognitive tests, biomarkers, or both. Cognitive assessments can be influenced by cultural, educational, and linguistic factors, while biomarker-based classifications, especially emerging blood biomarkers, may show variability across ethnic groups and lack validation in non-Western populations. These findings underscore the need for culturally adapted cognitive measures and biomarker validation in diverse settings.

Overall, we were pleasantly surprised by the success of our project, and we hope to recruit even larger samples in the future, and to analyze other neurodegenerative fluid biomarkers and the staging of various dementia subtypes. We are very hopeful that our work will contribute to improving clinical and biological adjudication of the accuracy of the diagnosis of AD and other neurodegenerative dementias in SSA, which will, in turn, decrease the potential diagnostic heterogeneity that might currently exist. Additionally, we only focused on participants with dementia without including other intermediary cognitive decline (e.g., MCI cohort), which could be seen as a limitation as well. The decision to exclude MCI was based on both funding limitations and the opportunity to investigate patterns of dementia that are well characterized in the Western world, as an opportunity to establish the validity of these techniques in SSA. With this success, we ultimately want to build a cohort of more diverse Congolese older adults to investigate many other fluid biomarker hypotheses tested in the West.

In conclusion, despite some limitations, the current study provides the first and preliminary patterns of dementia based on the biological definition of dementia and their cognitive and neuroimaging profiles in elderly adults with clinical dementia in Kinshasa/DRC. Future research should build on the methods and findings provided by our exploratory study to establish gold standard thresholds for different fluid biomarkers, the classification of various dementia subtypes based on these biomarkers, and the harmonization with clinical classification in probable AD and related dementia patients in SSA and DRC. Future research should also include cohorts of patients with intermediary status of cognitive decline, amnestic and non-amnestic dementia.

## Data Availability

The raw data supporting the conclusions of this article will be made available by the authors, without undue reservation.
